# Daily level predictors of impaired driving behaviors in young adults: Protocol design for utilizing daily assessments

**DOI:** 10.1371/journal.pone.0275190

**Published:** 2022-09-27

**Authors:** Brittney A. Hultgren, Katarina Guttmannova, Christine M. Lee, Daniela Acuna, Rachel L. Cooper, Jason R. Kilmer, Jennifer M. Cadigan, Brian H. Calhoun, Mary E. Larimer

**Affiliations:** 1 Center for the Study of Health and Risk Behaviors, Department of Psychiatry and Behavioral Sciences, School of Medicine, University of Washington, Seattle, Washington, United States of America; 2 Department of Psychology, University of Washington, Seattle, Washington, United States of America; PLOS (Public Library of Science), UNITED KINGDOM

## Abstract

**Background:**

Motor vehicle crashes remain a leading cause of death among young adults (ages 18–25) in the United States. Many drivers implicated in these crashes are under the influence of alcohol, cannabis, or the simultaneous use of alcohol and cannabis. Extremely limited research has assessed impaired driving behaviors and their predictors at the daily level. Perceived norms and motives to use substances have empirical support suggesting they may impact impaired driving-related behavior. Novel approaches to assess these associations at the daily level are needed and may inform future intervention and prevention programs.

**Objective:**

The goal of the current study is to utilize electronic daily assessments to assess driving under the influence of alcohol, cannabis, or simultaneous use and riding with a driver impaired by these substances to assess variability and predictors of these impaired driving-related behaviors at the daily level. This present manuscript details a protocol, measures, and a plan of analyses to assess how within-person differences in perceived norms and motives to use are associated with the likelihood of engaging in impaired driving-related behaviors.

**Methods:**

Participants include young adults in Washington State who report simultaneous use in the past month and either driving under the influence of alcohol, cannabis, or simultaneous use, or riding with a driver under the influence of both substances in the past 6 months. Individuals who verify their identity and meet eligibility requirements will complete a baseline assessment after which they will be scheduled for training on the daily assessment procedure via Zoom. Next, they will be invited to complete daily surveys on Thursday, Friday, Saturday, and Sunday every other week for 6 months and a 6-month follow up assessment. Analyses will utilize multilevel models with days nested within individuals.

**Results:**

The study is currently recruiting participants. A total of 192 participants have been recruited and 100 have completed the study protocol. Data collection is expected to be completed in Fall 2022.

**Conclusions:**

This study utilizes a novel design to assess impaired driving and predictors at the daily level among young adults at high risk of impaired driving-related behaviors. Findings will provide unique data that will shape the knowledge base in the field of social science and public health substance use research and that may be helpful for future prevention and intervention efforts on impaired driving.

## Introduction

The current paper details the protocol for an ongoing study assessing daily variability and predictors of impaired driving and riding with an impaired driver within young adults (aged 18–25), specifically with impairment by alcohol, cannabis, or simultaneous use of alcohol and cannabis. Motor vehicle crashes are the leading cause of death in young adults in the United States [1] and approximately 32% of young adult drivers involved in fatal crashes are impaired by alcohol [2]. Partly due to tremendous public health efforts, alcohol-impaired driving and its related injuries and fatalities have declined since the 1980s; however, these declines have stagnated since 2009 [3]. Additionally, with the legalization of medical and non-medical (or “recreational”) use of cannabis in many states [4], an increase in driving under the influence of cannabis, as well as simultaneous use, has been reported [5, 6]. Some research suggests that vehicle crash risk is almost 10% higher with simultaneous use compared to alcohol use alone [7]. Together, this suggests that novel approaches are needed to inform prevention and intervention efforts targeting impaired driving while under the influence of alcohol, cannabis, and simultaneous use.

The current study will assess impaired driving-related behaviors at the daily level, providing a novel examination of how day-to-day changes may impact the decision to drive impaired or ride with an impaired driver. Examination at the daily level is important because some individuals who engage in these risky driving behaviors will not do so every day, or every time they use alcohol and/or cannabis. Extremely limited work has focused on daily-level predictors of impaired driving-related variables that may illuminate why these high-risk behaviors occur on some days and not others. One study reported individuals are more likely to drive after drinking alcohol when they consumed more than what is typical for them, with a stronger association observed when subjective intoxication was lower (i.e., when participants perceived themselves to be less intoxicated than what is typical for them [8]. Another recent study assessing young adult who simultaneously use alcohol and cannabis reported participants were more likely to drive impaired and ride with an impaired driver on days when they engaged in simultaneous use compared to alcohol-only days [9]. These findings underscore that impaired driving-related behaviors are most likely impacted by within-person differences that fluctuate over time. However, no research has assessed daily-level factors that influence these impaired driving-related behaviors beyond that of actual substance use and perceived impairment. This paper provides details of a novel protocol used to assess driving under the influence of alcohol, cannabis and simultaneous use, riding with a driver impaired by these substances, and two factors that may affect these impaired driving-related behaviors at the daily level: motives to use and normative beliefs. These variables have been shown to be malleable by prevention efforts and intervention in the past (e.g., [10–12]), and this research could provide valuable implications for new interventions efforts aimed at preventing impaired driving.

### Motives to use

Motivations to use alcohol and cannabis are typically separated into several domains that are differentiated by whether they are internally or externally generated and positively or negatively reinforcing including: 1) *Social–*using to increase being social with others (external, positively reinforcing); 2) *Coping–*using to reduce negative feelings or emotions (internal, negatively reinforcing); 3) *Enhancement–*using to increase positive feelings or emotions (internal, positively reinforcing); 4) *Conformity–*using to “fit in” and reduce feelings of social isolation or rejection (external, negatively reinforcing) [13, 14]. A fifth motive, *Expansion*, relates to using a substance to increase perceptual or cognitive enhancement [15, 16]. While drinking and, to a lesser extent, cannabis use has shown associations with higher levels of social motives to use in young adults [17, 18], more evidence suggests coping motives are associated with experiencing consequences of alcohol and cannabis [19–22]. Specifically, one study showed a direct association between drinking to cope and a subscale of alcohol-related consequences defined as risky behaviors, which included a self-report of alcohol impaired driving [23]. However, higher reports across all five domains of motives were correlated with driving under the influence of cannabis [24].

This past research suggests between-person differences in alcohol and cannabis motives and their associations with driving under the influence exist. However, recent research suggests that motives for use may also vary within individuals over time [21, 25–28], and these more temporal motives may be more strongly associated with substance use and consequences than globally assessed motives. For example, a study that assessed general college semester motives to drink and event-level drinking motives for Spring Break found only event-specific motives, and not the general motives, were associated with alcohol use and consequences during Spring Break [29]. Taken together, this research indicates a need to understand how motives to use alcohol and cannabis at the daily level may be associated with both impaired driving and riding with an impaired driver. By understanding the effects of motivations to use on these risky outcomes, we may be able to better tailor prevention messaging and focus interventions on gaining skills to identify and address motivations with the strongest associations to impaired driving-related behaviors.

### Perceived descriptive norms

Perceived descriptive norms assess how much or how often individuals perceive their peers engage in a specific behavior [30, 31]. Similar to alcohol and cannabis use motives, perceived descriptive norms of use have long shown to have a significant associations with alcohol use [32, 33] and cannabis use [34–36], as well as consequences related to their use [37, 38]. Regarding impaired driving-related behaviors, several studies have indicated the more individuals believe their peers engage in alcohol-impaired driving, the more likely they are to drive under the influence of alcohol themselves [39–42]. Less research has assessed the association between perceived norms of driving impaired by cannabis use and engaging in these behaviors. A study by Whitehill et al. [43] indicated young adults who perceived their peers used cannabis and rode with cannabis impaired drivers were more likely to both drive under the influence of cannabis and ride with a cannabis-impaired driver. Another study reported perceived peer norms of cannabis use were associated with an increased likelihood of driving while intoxicated by alcohol, cannabis, and simultaneous use one year later [44]. This suggests a continued need to assess associations between normative perceptions and actual engagement in impaired driving-related behaviors. Additionally, other research suggests that norms can vary significantly within individuals across days [45], indicating assessing the association between norms and behaviors for impaired driving-related behaviors should be considered and examined at the daily level.

### Barriers in assessing impaired driving behaviors

Despite research indicating that assessing risky driving-related behaviors and potential risk factors at the daily level may be necessary to understand important relationships that could aid in prevention efforts [46], extremely limited research has done so. One reason that daily assessments have not been utilized more readily to assess impaired driving-related behaviors is the difficulty in assessing these behaviors.

A review of the alcohol-impaired driving literature shows a wide range of wording for questions assessing driving under the influence of alcohol. Overall, there are typically two ways researchers ask these questions. One way is to ask about subjective impairment. For example, how often or whether you have “drove while drunk on alcohol” [47], “…driven when you perhaps had too much to drink,” [48], or “under the influence of alcohol” [49] are all questions that used. Other instruments ask about driving after drinking in general or a specific number of drinks consumed, such as “how many times did you drive a car or other vehicle when you had been drinking alcohol,” [50] or “…after having 5 drinks in a row,” [51]. Both these options have merits but also limitations. A large limitation is that of social desirability. Alcohol impaired driving is an illegal activity that carries potential financial, legal, and physical injury repercussions and is widely viewed negatively in society [52]; as such, individuals may underreport these behaviors in self-assessments. Additionally, if driving under the influence is asked when impairment is subjective, the participant needs to think their impairment was enough to be dangerous to drive. This is problematic due to the research showing that, compared to individuals who do not drive impaired, those who do drive impaired are more likely to underestimate their blood alcohol concentration (BAC) and level of impairment [53], which can lead to underreporting of the behavior. If alcohol-impaired driving is assessed using number of drinks, researchers do not always ask the period over which participants consume alcohol, or how long it was after they drank that they drove. If these details are asked, it may be difficult for participants to answer in retrospective surveys with longer recall time periods (e.g., 6 months, 1 year).

Assessment of driving under the influence of cannabis provides bias issues similar to alcohol-impaired driving in respect to social desirability and relying on participants’ evaluation of their own impairment. Frequent cannabis users may experience tolerance and, as one recent meta-analysis found, experience less impairment in relation to individuals who use cannabis less often [54]. However, research also suggests certain neurocognitive tasks associated with impaired driving, slowed reaction time, are impeded for regular and occasional cannabis users after using cannabis [55] and that there may be considerable between-person differences in the effects of tolerance on impaired driving [56]. The diversity of cannabis products, their varying routes of administration, and unprecedented concentration of tetrahydrocannabinol (THC; i.e., the main active ingredient in cannabis [57]) make it extremely difficult to estimate impairment levels in a comparable way to alcohol. Based off laboratory assessments of the effects of THC, impaired driving can occur if driving took place within 2–6 hours after cannabis use (see [58] for review). A more recent meta-analysis of these studies suggests most periods of impairment will last 5 hours and dissipate within 7 hours of use [54]. The most recent guidelines from Fischer et al. [59] suggest driving can be impaired 6–8 hours after inhaling cannabis and that impairment can last 8–12 hours after use of edibles (i.e., oral ingestion). Again, asking participants to report on driving within a specific number of hours after using cannabis over longer assessment periods (i.e., 6 months; 1 year) has the potential to lead to underreporting due to recall bias and further justifies a daily assessment approach.

Another difficulty when examining substance use (especially polysubstance use) and potential consequences, such as impaired driving, at the daily level is assessing the order and overlap of impairment and events. In regard to use of alcohol and cannabis in the same day, there are times when such use is simultaneous, in that the effects of the two substances overlap, and there are times when it is concurrent, in that the substances are used in the same day, but not at the same time and their effects do not overlap [60, 61]. To be able to get more objective assessments of impairment by simultaneous use and subsequent impaired driving behaviors, the timing of use of each substance and driving behavior is needed. This need may suggest that momentary assessments are necessary; however, surveys to be completed in the moment while under the influence of alcohol and/or cannabis may have issues with missing or incomplete data, and participants should not complete assessments while in the act of driving. While advances in biomedical technology are bringing us closer to momentary assessments of impairment such as a wearable continuous alcohol monitors (e.g., BACtrack Skyn), these devices are still new, and they have known difficulties in data loss and translating transdermal alcohol content (TAC) to BAC [62–64]. Additionally, such devices are presently only available for alcohol use and will not determine use of or impairment by cannabis. It is anticipated that non-invasive biosensors will eventually be available to assess both alcohol and cannabis impairment. Meanwhile, with impaired driving contributing significantly to fatal motor vehicle crashes [3], there is an urgent need to assess and understand correlates of these risky driving-related behaviors to inform and improve intervention and prevention efforts. Therefore, the current study is utilizing a novel self-report daily assessment design that focuses on reported times of actual use to establish an estimated window of risk for impaired driving, as well riding with an impaired driver. This study is assessing young adults who are at high risk of engaging in these behaviors, and we provide details of the protocol for recruitment, retention, and assessment, as well as challenges and modifications to address them.

### Objective

The purpose of this paper is to detail the protocol for a daily assessment study designed to examine impaired driving behaviors and associations between these behaviors and perceived descriptive norms and motives to use alcohol and cannabis among young adults at high-risk of impaired driving-related behaviors.

## Methods

### Project overview

The current project aims to assess how factors of perceived norms and motivations to use alcohol and cannabis are associated with impaired driving and riding with an impaired driver at the daily level. Young adults (aged 18–25) who engage in simultaneous use and have a recent history of driving under the influence or riding with a driver under influence of simultaneous use of alcohol and cannabis are asked to complete daily assessments in a measurement burst design (that is, completing assessments Thursday through Sunday, every other week) for 6 months. These days were chosen for assessments due to young adult substance use mostly occurring on or around the weekends [65, 66] and higher rates of alcohol impaired driving on the weekends [67]. In these daily assessments, participants report on the previous day’s alcohol and cannabis use, motivations for using, perceptions of others’ use and engagement in risky driving behaviors. Each day, they also answer questions about their past-day transportation use. For all alcohol-, cannabis-, and transportation-use questions, participants are specifically asked about the time periods of use to establish overlap and windows of risk. This study was approved by the university’s institutional review board.

### Target population and eligibility

Original eligibility criteria included being between the ages of 18 and 25, currently residing in Washington State (WA), reporting simultaneous use at least once in the past 30 days, and reporting driving under the influence of simultaneous use of alcohol and cannabis or riding with a driver under the influence of simultaneous use at least once in the past 6 months. Recruitment started for the current study on March 15, 2021. At this time, many restaurants and other establishments still enforced restrictions due to the COVID-19 pandemic. Additionally, many young adults experienced several major environmental changes including virtual classes, job change or loss, and living back with parents or other family [68]. Several recent studies suggest a reduction in alcohol and substance use for young adults at the onset of the pandemic [69, 70]. These factors may have negatively impacted eligibility rates for the current study. Due to a lower than anticipated percentage of participants meeting eligibility criteria, as of September 8, 2021, participants are eligible if they report driving under the influence of simultaneous use or alcohol only or cannabis only (i.e., relaxing the eligibility criteria to include impaired driving by either substance instead of only simultaneous impairment) and meet all other previous eligibility criteria. Participants must be proficient in reading English to enroll. Participants are excluded if they do not meet the eligibility criteria, report they plan to be living outside WA for more than 1 month in the next 6 months, their mailing address is not within WA state, they do not correctly answer attention-check questions, or they fail the online CAPTCHA test (see below).

### Sample size and recruitment

This study anticipates recruiting a total of 400 participants and is currently actively recruiting. There are a total of 52 daily assessments for participants to complete. The sample size was based off simulation-based power analyses that were informed by parameter estimates obtained from similar intensive longitudinal data on young adult simultaneous use in Washington state [9, 71]. Power analyses estimated our ability to detect a daily-level association between enhancement motives and riding with an impaired driver on simultaneous use days in a logistic mixed effects model that accounted for the clustered nature of the data (20,800 person-days clustered within 400 participants) and the intra-cluster correlation (ICC) [72, 73]. Simulations were performed across varying effect sizes (odds ratios ranging from 1.10 to 1.28) and sample sizes (300–400 participants). Based off previous daily data collection, riding with a simultaneous use impaired driver was selected for the sample size calculation as it was the outcome with the lowest base rate. Thus, our estimates are conservative. In the data used to provide parameter estimates for the power analyses, riding with a simultaneous use impaired driver occurred on 12.6% of simultaneous use days and the effect size for the daily-level association between enhancement motives and riding with a simultaneous use impaired driver was indicated by an odds ratio of 1.28 (*OR* = 1.28, 95% *CI*: 0.95, 1.73). Thus, we assumed that riding with a simultaneous use impaired driver would occur on approximately 12.6% of sampled days (i.e., 6.5 of 52 days, on average), and we used an odds ratio of 1.28 as a starting point for the effect size. Using 500 simulations per condition, we estimated we would be sufficiently powered to detect associations with odds ratios greater than or equal to 1.15 with 300 or more participants.

Recruitment utilizes several techniques. The primary form of recruitment is through social media, including posting content and paid advertisements on Facebook, Instagram, TikTok, and Twitter. Unpaid social media posts are also uploaded to Facebook, Instagram, Twitter, and Reddit, and information on the study is provided on a research website for the university. Drawings for giveaways of e-gift cards for amounts ranging between $10–$25 for individuals to follow, like, and tag friends within comments of posts on Instagram and Twitter are also being used. The study also has a website to provide information on what participation involvement entails and the contact form (see below). Study team members are in contact with state and local stakeholders, community and wellness centers, and unions to ask them to share our social media posts, to share electronic information about our study, or to be sent physical recruitment flyers. COVID-19 has delayed posting of flyers in many public locations, but since WA has re-opened more flyers will be posted in libraries, community billboards in cafes and other locations. Flyers include phone and e-mail contact information for the research team as well as the URL and a QR code to complete the initial contact form. Lastly, individuals who have completed previous research studies within the center with which the current research team is affiliated with and have provided consent to be contacted for other research opportunities are being contacted via e-mail and/or phone to invite them to complete the screening survey. All recruitment materials and communication with potential and current participants explain the study as assessing young adult transportation and health behaviors.

### Contact form

Participants who are interested in enrolling in the study are directed to complete the initial contact form. This form asks participants to provide their contact information: name, mailing address, e-mail address, and date of birth. There are also checks to help ensure individuals who are completing the contact form are live participants (i.e., not robots) residing within WA who have not already participated. First, participants must pass a CAPTCHA image test to ensure they are a live person and not a robot attempting to complete the form automatically. If participants cannot complete the CAPTCHA, they cannot submit their contact form. Within the form, participants need to complete four attention-check items that ask question such as “Please select the Toyota model Camry from the list below.” If their mailing address is not located in WA state, they are not in the age-eligibility window [i.e., 18–25], or they answer an attention-check question incorrectly, participants receive a thank you message that lets them know they are not eligible at this time. Participants who have completed all requirements and initial eligibility checks will receive a message thanking them for their interest in participation and letting them know a research team member will be contacting them with information on the next steps.

### Phone verification

After successful completion of the contact form, participants are called by a study team member or an undergraduate/post-baccalaureate research assistant to confirm the participant’s name, date of birth, mailing address and email address. Participants who initially opt out of text messages in the contact form are asked if they would like to receive text messages or continue with only email notifications. Once verified, participants are emailed a link to the screening survey. This verification process was initially implemented because previous studies we have conducted with similar recruitment techniques encountered issues with numerous fake participants (i.e. robots) entering a study, and we have found this verification procedure to be mostly effective correcting this issue. Therefore, to allow for easier flow of participants from the contact form to screening survey, we have currently adjusted the protocol to allow potential participants to be emailed a link to the screening survey if they are not reached via phone, but their voicemail includes their name. We still have experienced some participants to pass through the phone verification who were determined to provide false information (e.g., older than 25, live outside WA state) and therefore have added an additional identity check during the virtual training (see below).

### Screening survey

After verification, individuals are emailed a personalized link that directs them to the research statement and the screening survey. The research statement includes information about the study, the screening survey, and the rest of the study procedures if they are eligible. After providing consent, participants are automatically directed to the screening survey. This survey takes approximately 10 minutes to complete and is used to confirm the rest of the eligibility requirements. This survey includes additional demographics, questions on participants’ alcohol, cannabis, simultaneous use, and other drug use, transportation availability and habits in the past 6 months, impaired driving-related behaviors in the past 6 months (e.g., alcohol-impaired driving, riding with a cannabis impaired driver), information on past driving tickets/citations, and other health risk and mental health behaviors and symptoms (i.e., sleep behavior, positive and negative affect, and anxiety symptoms). Due to potential changes in transportation related to the COVID-19 pandemic, we also ask participants to indicate if their use of different forms of transportation (e.g., public transportation, ride services) has changed due to the COVID-19 pandemic. Participants who meet eligibility requirements are immediately redirected to the Baseline survey.

### Baseline survey

Eligible participants directed to the Baseline survey will first receive a consent form detailing the next steps of the study procedure. Participants must provide consent prior to answering the Baseline survey questions. This survey takes approximately 25–35 minutes to complete, and it assesses more detailed questions about alcohol and cannabis use and consequences, perceived norms of peer use and impaired driving-related behaviors, motives of use, personality traits (i.e., impulsivity and conscientiousness), aggressive and distracted driving, transportation use and availability, impaired driving-related behaviors, and adverse childhood experiences. At the end of the Baseline survey, participants are directed to an online scheduler to schedule a virtual training session on the daily assessment protocol.

### Virtual training for daily assessments

**Fig 1:**
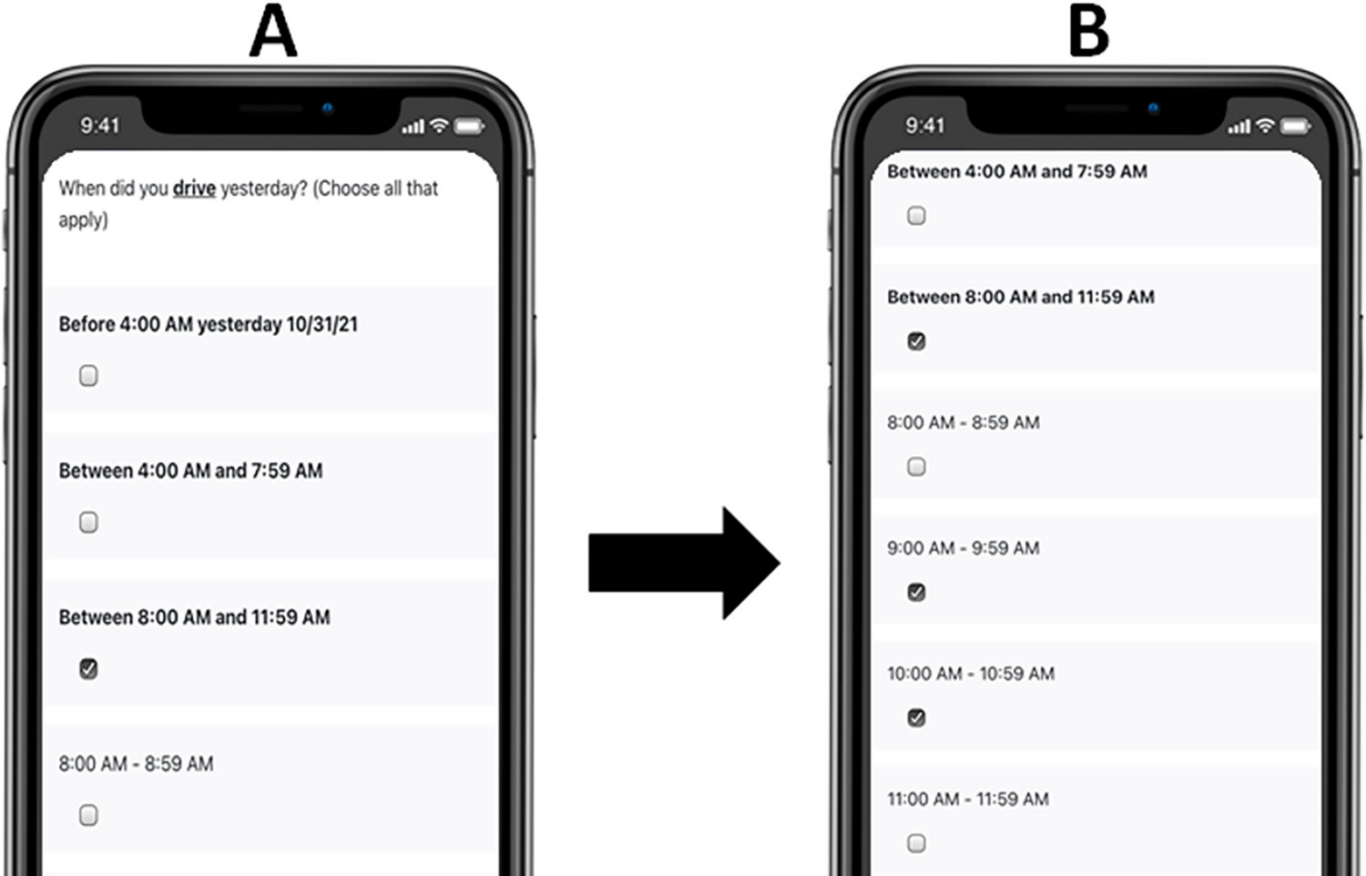
Example of daily measures assessing timing. Note: Participants first receive the screen shown in panel “A.” When a 4-hour period is chosen, below it 1-hour increment options appear as shown in panel “B.”.

Training sessions are one-on-one, facilitated by the study team, and typically take 15–20 minutes. These sessions occur over the HIPAA compliant Zoom platform. The purpose of the training is to orient participants further on when to complete the daily surveys, what questions will look like, and the payment schedule they should expect. First, participants are asked to verify their identity and residency by either showing their WA State identification or another form of identification and a piece of mail showing their address. Participants are trained on 1) how to measure a standard drink based on the definition by National Institute on Alcohol Abuse and Alcoholism [74], 2) our definition of cannabis/cannabis is, “any products with THC (including products with both THC and CBD),” and 3) our definition of vehicle is “car, light truck, or motorcycle” are explained. Due to COVID-19 pandemic-related restrictions, it is possible participants are having many interactions with other people virtually, instead of in person. Therefore, in the training sessions, we describe that we are defining a virtual interaction as “direct interaction with people virtually/online in real time” and describe some examples that qualify such as Zoom or Facetime calls and playing video games with an audio chat feature (such as Xbox Live); as well as examples that do not qualify such as scrolling through Instagram or Facebook, watching and sharing videos on TikTok, and text messaging with a friend. Participants are also shown screenshots of some of the assessment questions. A specific example is shown on how to answer questions about the timing of behaviors (see Fig 1 and further explanation in the Daily Assessment Procedure section). Lastly, participants are quizzed on their knowledge of the procedures by providing the participant with hypothetical scenarios and discussing with the study staff how they would provide answers in the survey. These scenarios are designed to assess their knowledge of our study definitions and how to determine the standard number of drinks. Participants are also provided with an opportunity to ask any additional questions about the study and are provided all the contact information and social media links again.

To increase participant study flow, we have created an optional recorded training for participants with whom we have difficulty scheduling the virtual one-on-one training. If scheduling attempts are unsuccessful (e.g., participant does not schedule a training within two weeks; participant has two or more missed scheduled training times), participants are sent a link to the training video and a quiz with 10 questions. Participants must answer 80% correct to move on to the daily assessment survey portion of the study. Participants are allowed to take the quiz multiple times until they achieve the score needed to move them forward, as its purpose was to train participants in daily assessment protocol and language.

### Daily assessment procedure and monitoring

Participants start their daily assessments depending on when they complete their virtual training. If they have a training session on Monday or Tuesday, participants start their surveys on the Thursday of that same week. If participants complete their training on Wednesday through Friday, they start completing daily assessments on Thursday of the following week. Participants complete surveys on Thursday, Friday, Saturday, and Sunday every other week for 6 months. On Wednesdays of an active assessment week, participants are sent a reminder text message or email (if they have not provided consent to text them) that they will be sent a survey invitation tomorrow. Wednesday reminders are sent at 4:00pm. Participants are sent a text message or email that includes the URL to the daily assessment survey on Thursday–Sunday at 10:00am with reminders at 12:00pm, 2:00pm, and 4:00pm if they do not complete the daily assessment. The survey is open from 10:00am to 5:30pm, but—to allow for completion time—participants are told the survey closes at 5:00pm. If a participant misses all four surveys in an assessment week, the study staff call them to verify they are receiving the invitations and that they are not having any technical difficulties with the surveys.

### Six-month follow-up assessment

After completing 6 months of the daily assessment procedures participants are invited to the 6-month follow-up survey. This survey is similar to the Baseline survey and assesses alcohol, cannabis, simultaneous use, and other drug use, alcohol and cannabis consequences, transportation availability and habits, impaired driving-related behaviors, and driving tickets/citations in the past 6 months. Participants also report on other health risk and mental health behaviors and symptoms (e.g. depressive symptoms), and impaired driving-related cognitions (e.g., norms, motives).

### Compensation

Participants can receive up to $281 for completing the study procedures. A $5 e-gift card is provided for completing the Screening survey, $25 for completing the Baseline survey, and $30 for completing the 6-month assessment. For the daily assessment procedures, participants receive $3 for each survey and a $5 bonus for completing all four surveys within one week. Therefore, participants can receive up to $17/week for each assessment week of the daily assessments.

### Daily assessment measures

#### Mood and affect

Participants are asked to rate how the current day and yesterday were on 5-point scale from Very bad (-2) to Very Good (2). They also complete the Positive and Negative Affect scale short form adapted for daily assessments [75].

#### Transportation

Participants are asked if they went anywhere yesterday. If they indicate they did, they are asked what forms of transportation they used out of the following list: 1) Public transportation (e.g., bus, light rail), 2) Taxi or cab, 3) Ride Service (Uber, Lyft), 4) Got a ride from someone such as a friend or family member (not a taxi or ride service), 5) Drove yourself 6) Rode a bicycle 7) Walked. For each type of transportation option that is chosen, participants are asked a follow-up question on the times they used the transportation. Participants see response options that are initially presented in 4-hour intervals. Once a 4-hour time interval is selected it, the assessment narrows and moves on to response options that are 1-hour intervals (see Fig 1). Participants who report getting a ride from someone are asked to indicate who they received a ride from with a “check all that apply” list that includes: 1) Parent or guardian, 2) Family member other than a parent or guardian, 3) Friend, 4) Significant other, partner, or spouse, 5) Other: _______. One question assesses the daily impact the COVID-19 pandemic has had on their transportation choices with response options ranging from 0 “Not at all” to 4 “Extremely.”

#### Substance use

Participants indicate “Yes/No” to whether they 1) drank alcohol and 2) used cannabis. If they report yes to both alcohol and cannabis/cannabis, participants are asked to respond Yes/No to “Did you use alcohol and cannabis at the same time, that is so their effects overlapped yesterday” to assess self-reported simultaneous use. Participants who indicate simultaneous use on the previous day report how much the feeling or effect of alcohol overlapped with the feeling or effect of cannabis using a 4-point scale that ranges from 0 “Not at all” to 3 “A great deal.” Participants are also asked to indicate which hours they drank alcohol and which hours they used cannabis yesterday by first responding to the same 4-hour intervals that are used to assess transportation use that, once selected, move to 1-hour interval windows. For alcohol use, participants are asked to report the number of drinks consumed for each hour they indicated they drank. Participants who reported cannabis used on the previous day are asked to indicate the ways they used cannabis yesterday from a “check all that apply” list that includes: 1) Smoked it, 2) Ate it, 3) Drank it, 4) Vaporized it (with a vape pen or e-cig), 5) Used it by dabbing and 6) Used it some other way.

#### Time-ordering of transportation and substance use

**Fig 2:**
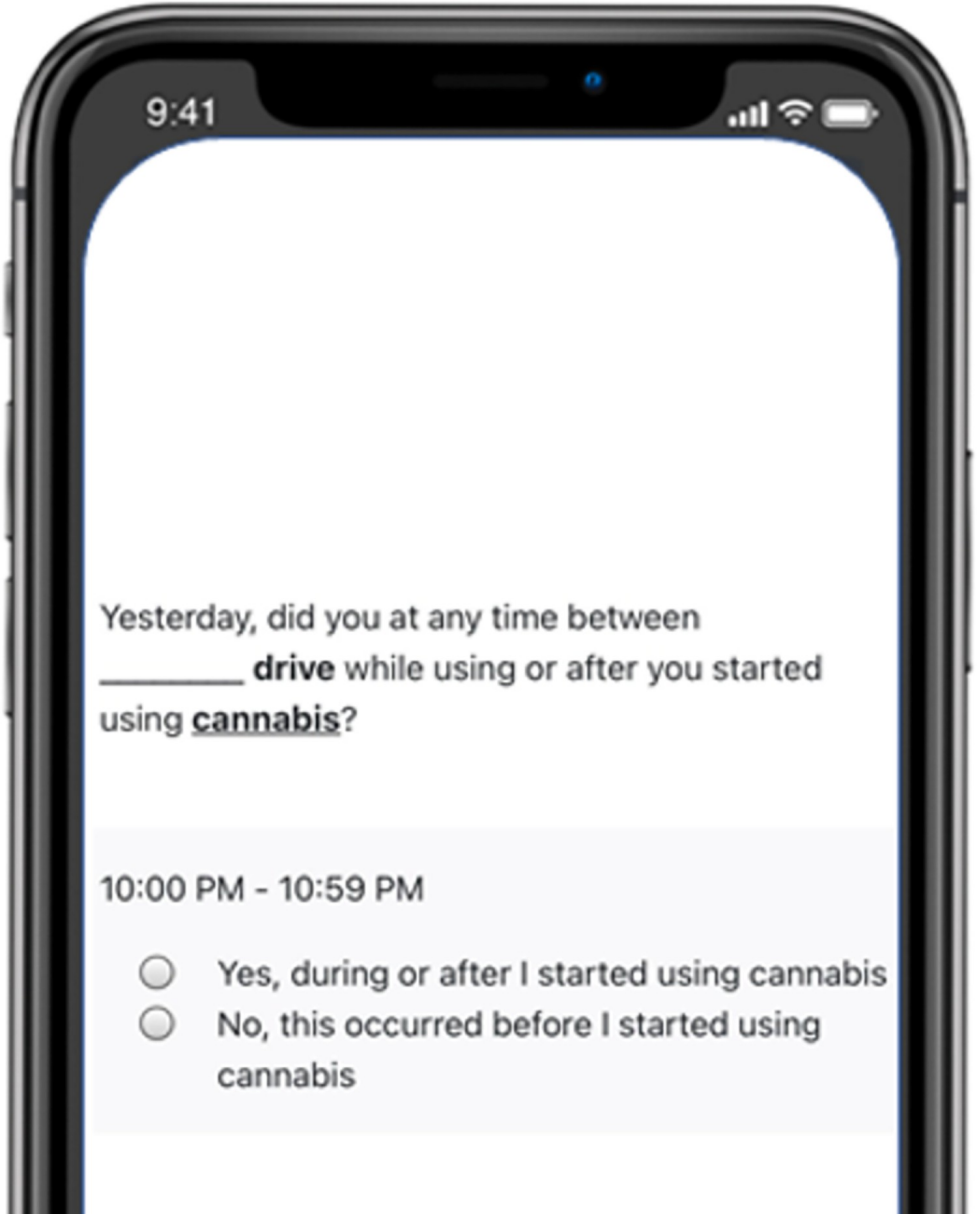
Example of question assessing overlapping transportation and substance use. Note: For every 1-hour period a participant chooses for both a transportation and alcohol and/or cannabis use question, they are provided with this follow-up question to determine when transportation occurred in relation to the substance use.

Since the focus of this study is to assess impaired driving behaviors, we need to be able to assess the time-ordering of participants’ substance use and transportation if they said they used both in the same 1-hour interval. For example, if a participant reports driving from 1pm–2pm and reports drinking from 1pm–2pm, it is not known if the participant started drinking before or after they were driving. To disentangle this, participants who have overlapping time periods of substance use (i.e., alcohol and/or cannabis use) and transportation use (e.g., driving, ride share, using a bicycle) are asked a follow-up question through which participants indicate whether the transportation occurred during or after they started using alcohol/cannabis or whether it occurred before they started using alcohol/cannabis (See Fig 2 for an example).

*Riding with an impaired driver*. Participants who report getting a ride from someone are asked to answer Yes/No to whether the driver: 1) Drank any alcohol before driving, 2) Drank 4 or more drinks within 2 hours of driving, 3) Used cannabis within 3 hours of driving, and 4) Used alcohol and cannabis within 3 hours of driving. This is asked specifically for each person a participant indicates that gave them a ride.

#### Motivations for alcohol and/or cannabis use

Participants who report using alcohol and/or cannabis on the previous day are asked the question “Yesterday, to what extent did you use alcohol and/or cannabis for the following reasons?” using response options that range from 0 “Not at all” to 4 “Extremely.” Participants report on 14 possible motives that were adapted from past research [13, 76–78]. Two items assess conformity motives (e.g., “So I wouldn’t feel left out”); seven items assess coping motives of which two assess social anxiety specifically (e.g., “To manage social anxiety”); two items assess motives for social reasons (e.g., “To make a social gathering more enjoyable”); two items assess enhancement motives (e.g., “To feel good”); and one item assesses use for an altered perspective, (e.g., “To alter my perspective or to think differently”), sometimes referred to as an expansion motive [15, 16].

#### Motivations to not use alcohol and/or cannabis

Participants who do not report any alcohol or cannabis use are asked “Yesterday, to what extent did you choose NOT to use alcohol and/or cannabis for the following reasons?” using response options that range from 0 “Not at all” to 4 “Extremely.” Participants report on 12 possible motives. Six of the items are from previous research [79, 80] and ask about motives related to work or school obligations that day, not having any one to drink or use cannabis with, not being able to get either substance, not having a desire to use either substance, and not usually using either substance on that day of the week. Five items have been used in other daily assessments and assessed needing money for other things, not wanting it to interfere with work/school today, having children around, having parents around, and other:_______ [76]. One additional item was added for the current study which assessed the motive “I needed to drive somewhere.”

#### Norms

Participants are asked three questions to assess perceived descriptive norms of substance use and eight questions to assess perceived descriptive norms of impaired driving and riding with an impaired driver. All norm questions prompt participants to “Think of the people you were around (either in-person or virtually/online) yesterday.” For substance use, participants are asked how many of these people they think were using or had used: 1) alcohol, 2) cannabis, and 3) alcohol and cannabis at the same time. Participants report on their perceptions of others around them driving impaired by reporting how many of these people they think drove 1) After drinking any alcohol, 2) After drinking 4 or more drinks in 2 hours, 3) Within 3 hours after using cannabis, and 4) Within 3 hours after using both alcohol and cannabis at the same time. To assess perceptions of others around them riding with an impaired driver, participants answered these four questions again but regarding how many of these people they though were a passenger of a driver under each of the four circumstances (e.g., After drinking any alcohol). Response options for all norm questions were on a 7-point scale: 0 people, 1–2 people, 3–5 people, 6–10 people, 11–25 people, 26–50 people, and 50+ people. A scale was chosen instead of an open response to reduce cognitive burden on participants [81], and these response choices were chosen to reflect a range of possible different size groups from only 1–2 other people all the way up to a larger party, or crowded bar/restaurant.

#### Context questions

Participants indicate how many people they were around (either in-person or virtually/online) yesterday using the same scale used to assess the norm questions that ranges from “0 people” to “50+ people.” They then have a checklist to indicate their relationship to the participants (e.g., parent(s) or guardian(s), friend(s), coworker(s)). Additionally, participants are asked to indicate if they worked a “graveyard” or 3^rd^ shift, such as working midnight to 8am. Participants who report not going anywhere the previous day (and therefore not using any transportation) are asked to indicate what they did yesterday from a “check all that apply” list that includes: 1) Attended school virtually or completed school work, 2) Worked from home, 3) Took care of a child or other family member, 4) Video chatted with friends or family members, 5) Used social media, 6) Played video games, 7) Completed chores or housework, 8) Watched TV or streamed movie(s) or TV show(s), 9) Napped, 10) Exercised, 11) Other:_______.

#### Sleep, exercise, and diet

Participants are asked additional questions related to sleep, exercise and diet when they indicate either 1) they did not travel or 2) they did not use alcohol or cannabis, they are asked questions on health behaviors. These additional questions are asked to make the survey length is approximately equal across days. Participants are asked six questions taken from the Consensus Sleep Diary [82] on their sleep last night including the time they went to bed, the time they got up that day, how long it took them to fall asleep, how much they were awake in the middle of the night, how long they were awake before they got out of bed, and the quality of their sleep the night before. Exercise the previous day is assessed using four questions from the daily adaptation of the Godin Leisure Time Exercise Questionnaire [83, 84]. Daily diet was assessed by asking the number of servings they had the previous day of 1) fruits, 2) vegetables, and 3) 8oz of sugary drinks such as soda.

#### Additional transportation risk behaviors

Participants who do not report using alcohol or cannabis are asked additional transportation-related risk behaviors. Again, this choice was made to make the survey approximately equal in length if participants did not use substances. Seat belt use was assessed for participants who did not use either substance but reported driving or riding in a car (either from a friend/family member, cab/taxi, or ride service). Participants report on how often they wore a seat belt while they were in car yesterday using the response options 0 “Never,” 1 “Sometimes,” and 2 “Every time.” Participants who report not using either substance but report driving are also asked to indicate Yes/No to the question “Did you read or send any text messages while driving yesterday?” and rate their drowsiness while driving using the response options 0 “Not at all tired or drowsy,” 1 “A little bit,” 2 “A moderate amount,” and 3 “Very tired or drowsy.”

#### Reasons for not traveling

To contextualize participants’ responses, if participants report not traveling anywhere the previous day, they are asked to respond to six questions on their reasons for not using transportation using a 5-point scale that ranges from 0 “Not at all” to 4 “Extremely.” The reasons assessed are 1) COVID-19, 2) Not having a transportation option available, 3) Not having money for transportation, 4) Responsibilities at home, 5) Low mood or feeling down, 6) Not feeling like going anywhere.

#### Arguments, disagreements, and stressful events

Participants who report 1) not traveling and/or 2) not using alcohol and/or cannabis are asked questions about arguments or disagreements they had the previous day, using a revised version of the Daily Inventory of Stressful Events (DISE; [85, 86]) Again, this choice was made to provide surveys of approximately the same length. Participants are asked to respond Yes/No if they experienced the following yesterday: 1) Did you have an argument or disagreement with anyone, 2) Did anything happen that you could have argued about but decided to let pass to avoid disagreement, 3) Did anything happen at school or work that most people would consider stressful, 4) Did anything happen where you live that most people would consider stressful, 5) Did anything happen to a close friend that most people would consider stressful, and 6) Did anything else happen to you that most people would consider stressful. For each of these six experiences that are endorsed, participants are asked to rate how stressful the experience was for them using a 4-point scale that ranges from 0 “Not at all” to 3 “Very.”

### Data analysis plan

Generalized linear mixed models (GLMMs) will be used to assess the main aims and address the nature of the nested data. Daily assessments (Level 1) will be nested within individuals (Level 2) to account for clustering of occasions within individuals and accommodate unequal observations per person [72, 73, 87]. Outcomes are assessed at the event level; therefore, binary distribution will be used, and findings will be expressed in terms of odds ratios (OR). Norms and motives will be assessed as both time-invariant and time-varying predictors of all impaired driving-related outcomes. Level 1 variables will be person-mean centered and Level 2 variables will be grand-mean centered. Models will be adjusted for sex, age, race/ethnicity, availability of safe alternative transportation, and estimated event-level alcohol use. Since the minimum legal age to purchase and use cannabis is 21 in WA, we will assess age both as a continuous covariate and with results stratified by those under 21 compared to participants 21 and older. These analyses will allow us to assess the within- and between-person variability across 6 months. Additionally, we will be able to assess whether both overall- and event-level perceived norms and motives to use are associated with increased odds of impaired driving-related outcomes.

## Results

As of June 22, 2022, we have had a total of 5,867 participants complete the initial contact form. Of those, 2,238 (38.1%) had their identity verified and completed the screening survey. A total of 264 (11.8%) participants were eligible. Of the eligible participants, 232 (87.9%) completed baseline and 200 (86.2%) were scheduled for a virtual training or completed the video training and quiz. Nine participants have dropped from the study—six prior to completing any daily assessments, two shortly after starting daily assessments, and one after completing 36 of 52 daily surveys. Seven participants were removed from the study because it was determined they provided false information and did not live in Washington. Five of these individuals were removed prior to completing any daily assessments, the other two shortly after starting daily assessments. All data from participants who provided false information and daily assessment data for the two participants who dropped shortly after starting daily assessments were removed. As of June 22, 2022, 192 participants started daily assessments. Of the 8,152 potential daily assessments that could have been completed on or prior to June 22, 2022, 81.2% were completed and an additional 2.3% have partial data. The racial and ethnic composition of the 192 daily assessment participants is: 64.1% White, 21.4% Asian/Asian American, 3.6% Black/African American, 1.0% Native Hawaiian/Pacific Islander, 0.5% American Indian/ Alaskan Native, 6.3% Multiracial, 3.1% Other Race, and 10.4% Hispanic or Latino/a. Enrolled participants are an average 21.97 (SD = 1.99) years old, 70.8% reported female as their birth sex and 29.2% reported male as birth sex. Gender identification was 63.5% women, 28.1% men, 1.0% trans men, 5.7% genderqueer/ gender non-conforming, and 1.5% other gender not listed or no answer. Sexual orientation was 57.3% straight/heterosexual, 22.9% bisexual, 2.1% gay, 2.1% lesbian, 4.2% questioning, 7.8% queer, and 3.6% other. A total of 59.4% participants are college students and 43.2% have received a 4-year degree. As of June 22, 2022, 115 participants were invited to the 6-month follow-up assessment and 100 have completed. Recruitment and enrollment are anticipated to continue until the target sample size of 400 or on December 1, 2022, whichever comes first.

## Discussion

### Significance of future findings

The current study will assess how both perceived norms of impaired driving behaviors and motives to use alcohol and cannabis are associated with impaired driving-related outcomes, including driving under the influence of alcohol, cannabis, and simultaneous use and riding with a driver under the influence of these substances. The protocol detailed in this paper provides information on our innovative approach in assessing within-person and between-person associations potentially related to impaired driving outcomes. Impaired driving-related behaviors and their predictors have extremely limited research focused on daily-level associations [8, 88]. Therefore, the current study will provide unique informative data that will shape the knowledge base in the field of social science and public health substance use research and that may be helpful for future prevention and intervention efforts aimed at reducing harms related to substance use and advancing traffic safety and preventions of impaired driving-related behaviors.

The current study utilizes daily assessments with detailed timing questions as opposed to momentary ecological assessments because of the limitations related to completing assessments in the moment while under the influence [89, 90]. This detailed time assessment will allow us to determine impairment with less subjectivity than self-report of impaired driving and will provide insight on how often young adults may be driving impaired without judging themselves as impaired.

### Past and current challenges

This study has had several challenges to overcome. The largest challenge has been from the COVID-19 pandemic. Recruitment was originally planned to start in summer of 2020; however, due to lockdown restrictions and numerous closures in Washington State, recruitment was delayed. It was anticipated that many young adults would not be driving or traveling at all during this time due to these restrictions including closures of businesses and a full ban of indoor dining. The Governor’s decrees and changes of state and local restrictions were closely monitored, and it was decided to start recruitment when the indoor dining ban was lifted across most of the state. Since research on young adult drinking at the onset of the COVID-19 pandemic suggested a reduction in drinking [69, 70], there were still concerns that our eligibility percentages would be lower than initially anticipated. The rate of individuals screening in were tracked, and in early September (approximately 6 months after the study’s launch) it was decided to open the eligibility criteria to allow young adults with reports of any impaired driving by alcohol, cannabis, or simultaneous use (instead of only under the influence of simultaneous use) to screen into the study. Another current challenge the study is experiencing is the percentage of participants who identify as female compared to those who identify as male. This is a common issue among health behavior research—that more females participate than males [91]—and is also indicative of the gender distribution of individuals who engage in social media, particularly Instagram, from where a large percentage of our participants are recruited. Survey research suggests 44% of women, but only 36% of men, in the United States use Instagram [92]. To address this, we are utilizing social media outlets that have a larger percentage of male users, specifically Reddit, and we have planned in-person recruitment strategies in the future.

### Limitations

While the current study provides an innovative way to assess impaired driving-related outcomes and its projected findings may provide information that significantly improves intervention and prevention efforts, it is not without limitations. First, the sample consists of young adults who self-report either driving under the influence of alcohol, cannabis, or simultaneous use, or riding with a driver under the influence of simultaneous use of both substances. Thus, the findings may not be generalizable to individuals who engage in these behaviors but do not self-report them, nor to individuals who have never engaged in these behaviors. Future research is needed on what factors influence the first engagement in these behaviors, as previous research has repeatedly shown high associations between driving impaired in the past and future impaired driving [93, 94]. Second, the sample is specific to young adults in WA state, and additional research will be needed to determine if there are differences among other age groups—both older and younger than 18–25—and within other states with differing cannabis legalization statuses. Lastly, this data is being collected during the COVID-19 pandemic with variability occurring in businesses (including bars, music venues, and night clubs) being open, their hours of operation, and their capacity. As outlined above, while care was taken to ameliorate the obvious effects of the COVID-19 pandemic, it is unclear how these business restrictions and regulations, along with other changes occurring during the COVID-19 pandemic, will impact the overall findings, as is the case with many data collected during this time.

## Conclusions

This study will provide much needed information for prevention and intervention efforts on impaired driving behaviors. The intensive daily data collected will allow for innovative examination of potentially malleable predictors. This study is a cooperative agreement with CDC’s National Center for Injury Prevention and Control (NCIPC), and the scientific collaborators and scientific program officers have provided regular support and oversight that has been invaluable to the progress and success of the project. Additionally, the project has both current and future plans for involving additional partners at the state, region, and community level. We have received feedback on initial measures and are having continual open conversations about recruitment and current and future programs and initiatives to reduce impaired driving behaviors. In an effort to quickly translate research findings to “real world” settings, our team will be meeting with several stakeholders in Washington who are involved in impaired driving prevention to understand their successes and challenges, lessons learned that are useful for the present study, and how results from this research could be most helpful for their programs and prevention efforts. Once concluded, this project will provide reports on the findings of these results and the implications they may provide for these existing initiatives to stakeholders. Thus, the current project has potential for real-world impact on impaired driving behavior through this rapid dissemination of findings to partners, coalitions, and other key stakeholders.
